# A novel optical model of the experimental transmission spectra of nanocomposite PVC-PS hybrid thin films doped with silica nanoparticles

**DOI:** 10.1016/j.heliyon.2020.e04177

**Published:** 2020-06-09

**Authors:** Qais M. Al-Bataineh, A.M. Alsaad, A.A. Ahmad, Ahmad Telfah

**Affiliations:** a Department of Physical Sciences, Jordan University of Science & Technology, P.O. Box 3030, Irbid 22110, Jordan; b Leibniz Institut für Analytische Wissenschaften-ISAS-e.V, Bunsen-Kirchhoff-Straße 11, 44139 Dortmund, Germany

**Keywords:** Materials science, Materials chemistry, Nanotechnology, Optics, Polyvinylchloride (PVC), Polystyrene (PS), Silica nanoparticles (SiO2 NPs), Hybrid coating, Sol gel, Optical properties, Optical band gap energy, Hydrophobicity

## Abstract

We propose a novel derived optical model fitted to the experimental transmittance of PVC-PS hybrid thin films doped with Silica nanoparticles. The films are synthesized using a simple dip-coating method. The model has successfully interpreted the experimental spectral behaviour of transmittance of amorphous semiconductors and dielectric thin films. Interestingly, our model reproduces the optical parameters of the investigated thin films in good agreement with those predicted by Tauc plot. The great advantage of the proposed model over other models lies in its ability to explain the correlations between the film thickness and the optical bandgap. Furthermore, we investigate the structural, physical, and optical properties of PVC-PS- SiO_2_ thin films, in relevance to the silica percentage content. XRD measurements show that the as-prepared polymeric thin films are amorphous. In addition, SEM micrographs indicate that silica nanoparticles are well dispersed on the surface of the PVC-PS thin films with an average diameter of 100–400 nm. The effect of annealing parameters is also investigated to optimize the projected water contact angle of PVC-PS- SiO_2_ thin films. At annealing temperature of 2000°*C*, films become hydrophobic. The transmittance T% of the PVC-PS thin films is found to be about 83% in the visible region. The T% enhances to 90% upon adding silica NPs into PVC-PS polymeric thin films. Obtaining coatings with high transmittance is of crucial importance for several optoelectronic and photonic applications.

## 1 Introduction

The organic-inorganic hybrid optical materials are of great technological values. The elucidation between the properties of inorganic elements and polymer matrices may lead to hybrid compositions of astonishing properties. Furthermore, polymeric nanocomposites constitute an important class in the field of applied materials science technology due to their attractive properties [1, 2]. Polymer composites exhibits various interesting optical properties, such as high/low refractive index, tailored absorption/emission spectra and strong optical nonlinearities. Such rare properties make hybrids eligible for potential optoelectronic applications [3, 4]. The incorporation of inorganic nanoparticles (NPs) into polymers may enhance the electrical, optical and mechanical properties [5, 6] of the resulting nanocomposites. Silica-based inorganic–organic hybrids synthesized using sol-gel technique are being eminently employed in several technological applications at moderate temperature and pressure [7, 8, 9, 10]. Transparent hybrid such as tetraethoxysilane or tetramethoxysilane (TEOS or TMOS) that are free of phase segregation are synthesized properly at controlled conditions [11].

Recently, PVC polymer is one of the most multipurpose and multifunctional material worldwide. They have been used in the form of composites, blends and copolymers to outfit a diversity of tenders including flexible electronics [12, 13, 14, 15]. The flexible electrical circuits require specific mechanical properties at relatively high temperatures. Manipulation of PVC mechanical and elastic properties can be achieved by blend technology [16, 17] where PVC is mixed with other miscible or semi miscible polymer. In this study, PVC is mixed with amorphous polystyrene (PS) owe to its attractive properties such as, it is high transparency, very high electrical resistance and low dielectric loss. Outstanding mechanical properties including, hardness, stiffness and fragility [18].

In this work, PVC-PS polymer and PVC-PS-SiO_2_ NPs hybrid thin films with silica NPs contents ranging from zero to 10 wt. % are deposited on the glass substrate by dip coating sol-gel technique. Structural properties, surface morphology, water contact angle and optical properties such as transmittance, reflectance, optical constants, dielectric functions and optical band gap energy are measured, characterized and interpreted. We compared the optical band gap energy values obtained from our newly derived model with those obtained using absorption coefficient and Tauc plot methods. The main advantage of our model is that it can be used successfully to simultaneously determine five major optical constants. Mainly, the energy of band gap, thickness of film, the difference in bonding–antibonding energy states and the lifetime (τ) of the electrons involved in the optical transitions. The model fits the experimental transmission as a function of wavelength T(λ) by combining the five parameters of amorphous thin film materials. In addition, we compare the refractive indices of the prepared PVC-PS-SiO_2_ nanocomposite thin films obtained using the newly derived model with Sellmeier oscillator model.

## 2 Experimental details

### 2.1 PVC-PS polymer composite solution preparation

Polyvinylchloride (PVC) powder, Polystyrene (PS) powder, Tetrahydrofuran (THF) and Tetraethoxysilane (TEOS) are ordered from the leading, high technology Sigma-Aldrich company. The solutions are prepared by independently dissolving 0.3 g of PVC and 0.3 g of PS sequentially in 30 ml of pure THF at room temperature to form solutions A and B, respectively. The solutions A and B are then sonicated for 6 h to enhance the homogeneousness. The PVC-PS solution (solution C) is obtained by mixing solutions A and B in a 1:1 volume ratio using magnetic stirrer for 6 h. The solution is then filtered using 0.45 μm Millipore filter before dip coating on the glass substrates.

### 2.2 PVC-PS-silica nanocomposite solution preparation

The PVC-PS-silica nanocomposite solutions are prepared by mixing varying amount of TEOS (mass ratio from 0% to 10%) with the homogeneous PVC-PS solution (solution C) to yield silica network in PVC-PS matrix. The mixed solution is stirred for 2 h. Measured amount of water with HCl is added to carry out hydrolysis and condensation of TEOS, where HCl acts as a catalyst in the sol-gel process. The solutions are finally stirred for 5 h and each solution is poured into a separate petri dish. The final solution is then filtered using 0.45 μm Millipore filter before dip coating on the glass substrates [19].

### 2.3 Nanocomposites thin film fabrication

Before the dip coating, glass substrates are sonically cleaned in acetone, distilled water, and ethanol. The substrates are then dried by exposing it to a stream of blowing oxygen gas. The PVC-PS-silica nanocomposite solutions are deposited as a thin layer on glass substrate using dip-coating technique for 1 h forming nanocomposite thin films with average thickness of 300 nm. The nanocomposite thin films are obtained by allowing the solvent to evaporate overnight at 25 °C.

### 2.4 Characterization

The X-ray diffraction measurements are executed using (Rigaku Ultima IV) at our institute. The average thickness of the as-grown PVC-PS-SiO_2_ NPs thin film is measured is measured to be 300 nm using Scanning Electron Microscopy (SEM, Quanta FEG 450) images obtained at our nanotechnology facility. The room-temperature transmittance and reflectance are obtained using a Double-Beam UV–vis Spectrophotometer (U-3900H).

## 3 Results and discussion

### 3.1 Optical characterization of nanocomposites thin films

The key optical parameters and optoelectronic behavior of PVC-PS and PVC-PS-silica nanocomposite thin films for various silica NPs concentrations are deduced and interpreted by analyzing the transmittance T%(λ) and reflectance R%(λ) spectra in the spectral range 250–700 nm. It is worth mentioning that optical properties are sensitive to synthesis methods, morphology of surfaces of thin films, and doping levels [20].

**Figure 1:**
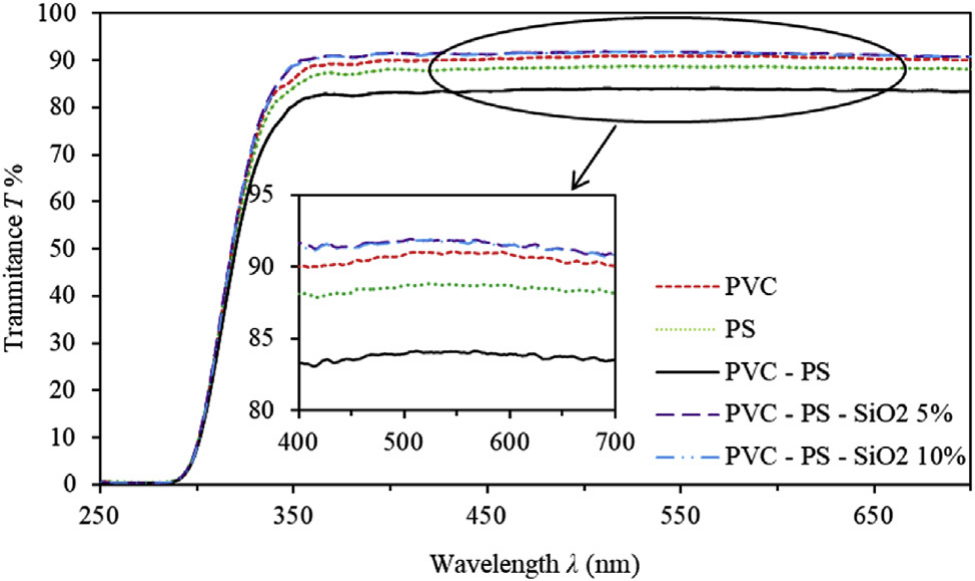
The transmittance spectra of PVC, PS, PVC-PS and PVC-PS-silica nanocomposite thin films with 5% and 10% concentrations of silica NPs.

**Figure 2:**
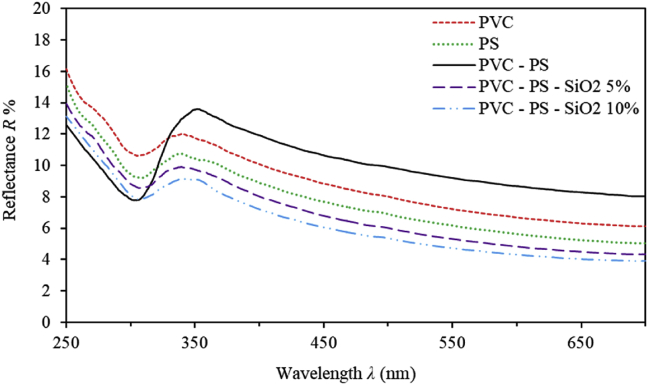
The reflectance spectra of PVC, PS, PVC-PS and PVC-PS-silica nanocomposite thin films with 5% and 10% concentrations of silica NPs.

The optical transmittance and reflectance spectra are measured at room temperature. Figures 1 and 2 depict the transmittance and reflectance spectra of PVC-PS and PVC-PS-silica nanocomposite thin films containing 5% and 10% silica NPs compared to the transmittance and reflectance spectra of PVC and PS thin films. To elucidate the dependence of optical behavior of as-prepared thin films, we analyse T% and R% spectra in two distinct main spectral regions. The PVC-PS and PVC-PS-silica NPs thin films exhibit an absorption edge in the high-energy region (*E ≥* 3.5 eV or λ ≤ 350 nm). Figures 1 and 2 indicate that as-grown thin films have relatively low transmittance and higher reflectance in this region. However, thin films exhibit high transparency (T%>80%) and negligible absorbance in the visible region (*E ≥* 3.5 eV, λ ≥ 350 nm). Interestingly, the absorption region is observed to be shifted into the blue region as the silica NPs content in PVC-PS thin films is constantly increased. This shift could be attributed to the rearrangement of film's constituents and the increase in the disorder, number of vacancies, crystallite size and agglomerations of atoms caused by introducing more silica NPs into polymeric matrix. Such significant variations of the film's features cause a considerable increase of the optical band gap energy. Quantitively, individual PVC and PS thin films are highly transparent in the visible region exhibiting T% of 90% and 88%, respectively. However, transmittance of PVC-PS hybrid thin film is only 83% indicating a decrease of transparency upon mixing PVC with PS. Fortunately, addition of silica NPs to PVC-PS matrix enhances the transparency. Our results indicate that PVC-PS-silica nanocomposite thin films containing 5% and 10% silica NPs have high values of transmittance of about 91% and 92%, respectively. This tendency could further be enhanced as the content of silica NPs is increased in the films. This trend could be explained in terms of the increase in the value of the optical band gap and the fact that silica NPs are strongly antireflective [21, 22, 23]. Therefore, nanocomposite thin films based on PVC-PS-silica hybrid could be used as anti-reflectance coating. Obviously, reflectance of undoped PVC-PS thin films is found to be about 10% at λ = 500 nm. The PVC-PS-SiO_2_ hybrid exhibits a decreasing reflectance in the visible region as more silica NPs are introduced in PVC-PS matrix.

**Figure 3:**
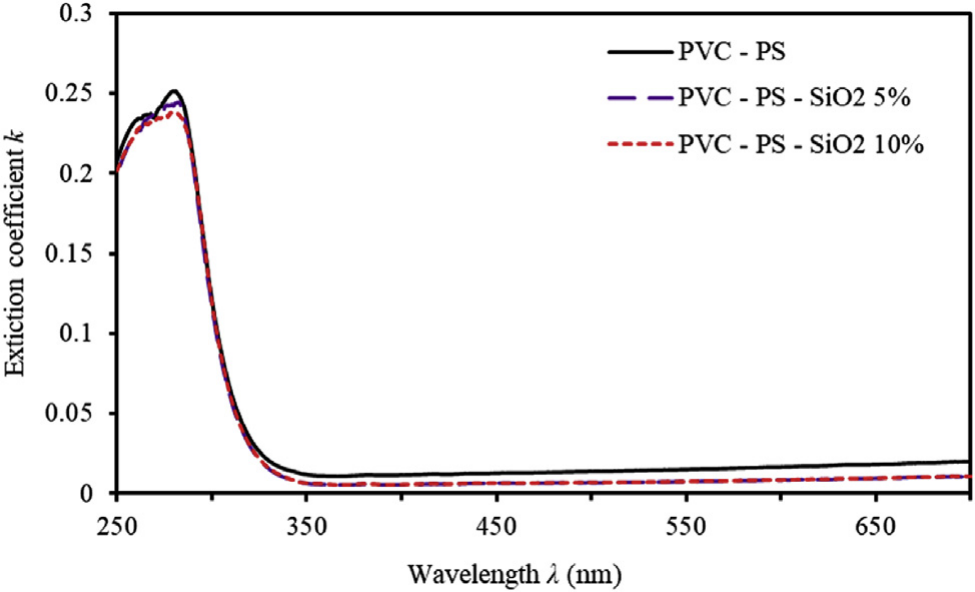
The extinction coefficient (k) of PVC-PS and PVC-PS-silica nanocomposite thin films with 5% and 10% concentrations of silica NPs.

Complex refractive index (N=n+ik) of thin films provides an effective instrumental tool to analyse and interpret the optical behaviour of thin films [24]. The extinction coefficient (k) signifies the absorption loss when light transmits through thin films. The average thickness (d) of films is measured using SEM micrograph and found to be around 300 nm. The extinction coefficient (k) is deduced easily as;

(1) k=αλ/4π

where α is the absorption coefficient defined by α=(1/d)ln(1/T) [25]. Figure 3 shows *k* as a function of the wavelength of incident light. Our results indicate that *k* of un-doped polymerized thin films decreases drastically and abruptly as it approaches zero in the wavelength range (260nm≤λ≤350nm). As the doping level of SiO_2_ NPs incorporated into PVC-PS polymeric matrix is increased, *k* is further decreased demonstrating that PVC-PS-silica nanocomposite thin films display no energy loss due to absorption and/or scattering. The change in *k*-value is within the order of 10^−3^ for different doping levels confirming good transparency. This behavior was reported for several other amorphous semiconductor thin films as found by Mott and Davis [26, 27].

Measuring refractive index (n) of thin films accurately is critical for designing optical components of optical devices [24, 28, 29]. In terms of reflectance and extinction coefficient *k*, it can be expressed as,

(2) $$n=\left(1+R/1-R\right)+\sqrt{\left(4R/{\left(1-R\right)}^{2}\right)-k^{2}}$$

**Figure 4:**
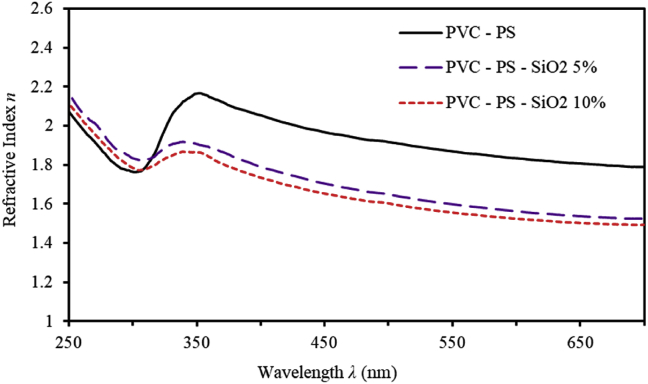
The refractive index (n) of PVC-PS and PVC-PS-silica nanocomposite thin films with 5% and 10% concentrations of silica NPs.

The resultant *n* spectra are displayed in Figure 4. In the high-wavelength regions, *n* spectra demonstrate normal dispersion. However, *n* spectra are characterized by anomalous dispersion in the low-wavelength regions. Furthermore, in the high-absorption regions (λ<350nm), *n* exhibits higher values. This could be interpreted rationally by the resonance effect prompted between the frequency of incident photon and the plasma frequency. Thus, electrons of PVC-PS-silica nanocomposite thin films are coupled to the oscillating electric field in this region. Beyond λ≥350nm, *n* decreases sharply demonstrating significant normal dispersion. Un-doped PVC-PS thin films have *n* values in the range (1.7–2.1) as λ varies in the range (350nm≤λ≥700nm). A further decrease of *n* of PVC-PS-SiO_2_ thin films is observed as the concentration of silica NPs increases.

In the high-wavelength region, the average oscillator wavelength $\left(\lambda_{0}\right)$ and oscillator length strength $\left(S_{0}\right)$ parameters are related to the refractive index n and the square of wavelength $\lambda^{2}$ as formulated by Sellmeier's optical mode [30],

(3) $$n^{2}-1=\left(S_{0}\lambda_{0}^{2}\right)/\left[\left(1-\lambda_{0}^{2}\right)/\lambda^{2}\right]\;$$

**Figure 5:**
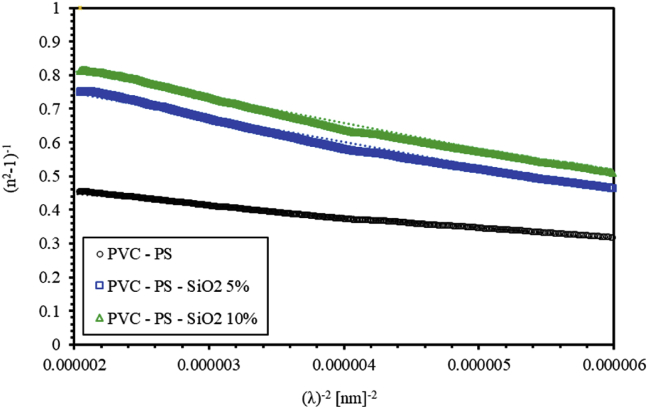
The plot of refractive index factor ${\left(n^{2}-1\right)}^{-1}$ versus the inversed wavelength squared ${\left(\lambda \right)}^{-2}$ of PVC-PS and PVC-PS-silica nanocomposite thin films doped with 5% and 10% of silica NPs.

Figure 5 shows the Plot of ${\left(n^{2}-1\right)}^{-1}$ versus $\lambda^{-2}$ of the as-grown doped polymerized thin film samples. The values of $S_{0}$ and $\lambda_{0}$ can be calculated from the slope $1/S_{0}$ and the intercept on the $\left(1/S_{0}\lambda_{0}^{2}\right)$ vertical axis. The obtained parameters are given in Table 1. As can be clearly seen from Figure 5 and Table 1, $\lambda_{0}$ value increases from 255.14 nm for undoped thin film to 285.63 nm for polymerized thin films doped with 10% of silica NPs. On the contrary, $S_{0}$ value decreases from $2.982*10^{-5}$ to $1.297*10^{-5}$ as silica NPs concentration increases from 0% to 10%. Figure 5 shows that the refractive index for our samples at higher wavelength follows Sellmeier's dispersion relation as expected for any optical material.

**Table 1:** Sellmeier optical parameters of PVC-PS and PVC-PS-silica nanocomposite thin films with 5% and 10% concentrations of silica NPs.

Parameter	Symbol	PVC-PS	PVC-PS-5%silica	PVC-PS-10%silica
Average oscillator wavelength (nm)	$\lambda_{0}$	255.14	285.63	282.47
oscillator length strength	$S_{0}*10^{-5}$	2.982	1.374	1.297

**Figure 6:**
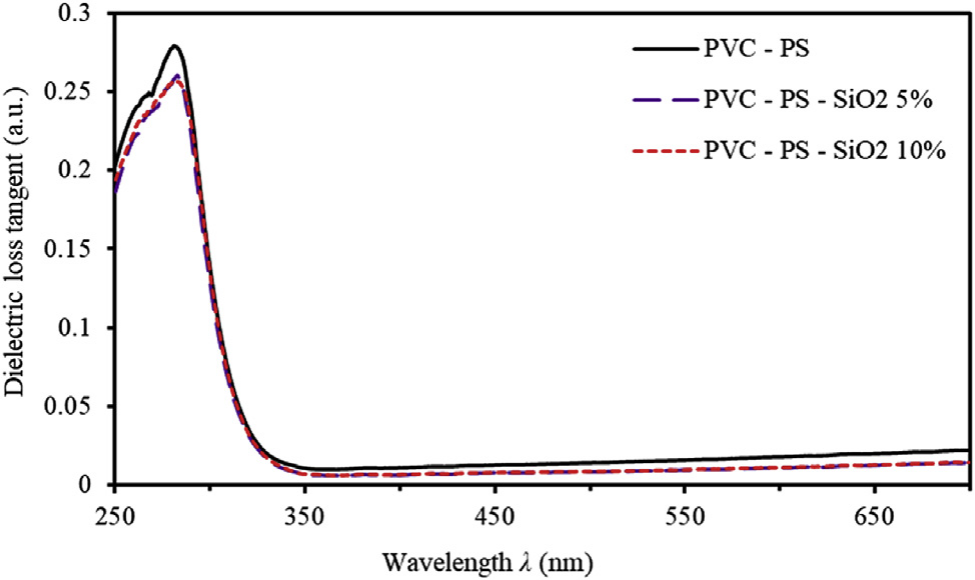
Dielectric loss tangent (tanδ) for PVC-PS and PVC-PS-silica nanocomposite thin films with 5% and 10% concentrations of silica NPs.

At a particular frequency, dielectric loss tangent (tanδ) represents the phase difference occurs due to the loss of the energy [31]. The tanδ parameter can be calculated in terms of the real ($\varepsilon^{'}$) and the imaginary ($\varepsilon^{''}$) parts of the complex dielectric function, as given by [4, 32],

(4) $$\tan\;\delta =\frac{\varepsilon^{''}}{\varepsilon^{'}}$$

where $\varepsilon^{'}=n^{2}+k^{2}$ and $\varepsilon^{''}=2nk$ [33]. Figure 6 shows tanδ of PVC-PS and PVC-PS-silica nanocomposite thin films containing 5% and 10% concentrations of silica NPs. Obviously, high-energy regions are characterized by high values of tanδ. As the wavelength increases, tanδ decreases abruptly. In the low-energy region (E<3.18eV or λ>390nm), tanδ assumes constant values approaching zero for all SiO_2_ NPs doping levels, as shown by Figure 6. In the visible region, tanδ increases monotonically. This trend could be attributed to the fact that tanδ is directly proportional to *k* and inversely proportional to *n*. Unambiguously, this is a consequence of the normal dispersion phenomena in this region. The peaks of tanδ are absent in the entire spectral range presumably due to the hopping of charge carriers [34, 35] as reported by Mott and Davis [26, 27].

### 3.2 Optical absorption studies, band gap energy and Urbach energy

**Figure 7:**
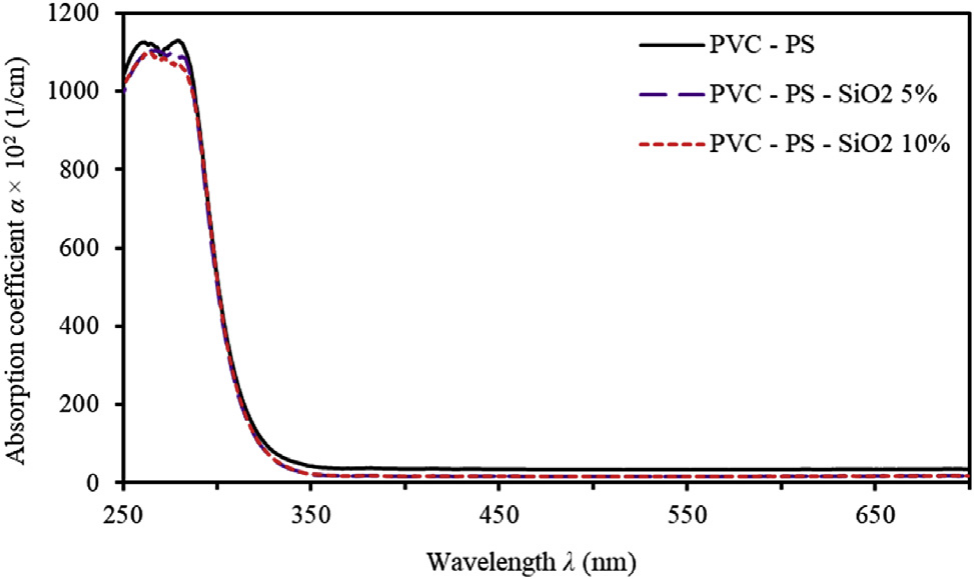
Absorption Coefficient (α) spectra for PVC-PS and PVC-PS-silica nanocomposite thin films with 5% and 10% concentrations of silica NPs.

We measure and characterize important optical parameters such as, the absorption coefficient (α), optical band gap ($E_{g}$), Urbach energy ($E_{U}$), Steepness parameter (σ) and electron-phonon interaction energy ($E_{e-p}$) [36, 37, 38]. Absorption coefficient as a function of wavelength (λ) is defined by [25]:

(5) α=(1/d)ln(1/T)

where, d is the thickness of the film measured by SEM micrograph and found to be 300 nm and T is the transmission value as a function of wavelength (λ). Figure 7 shows the absorption coefficient spectra of PVC-PS & PVC-PS-silica nanocomposite thin films contain 5% and 10% concentrations of silica NPs. Figure 7 shows that as the silica NPs are introduced in thin films, the absorption edge is observed in the visible region and found to be shifted into blue region. This may be attributed to several factors such as, rearrangement of film constituents, increasing in the disorder, increasing of the vacancies and increasing of the grain size and agglomerations of atoms. Thus, a noticeable increase in the band gap energy is observed. In this work, we study optical band gap energy of nanocomposite thin films using two different approaches. Mainly, Tauc plot and a newly derived model presented in this paper.

#### 3.2.1 Band gap energy using Tauc plot

**Figure 8:**
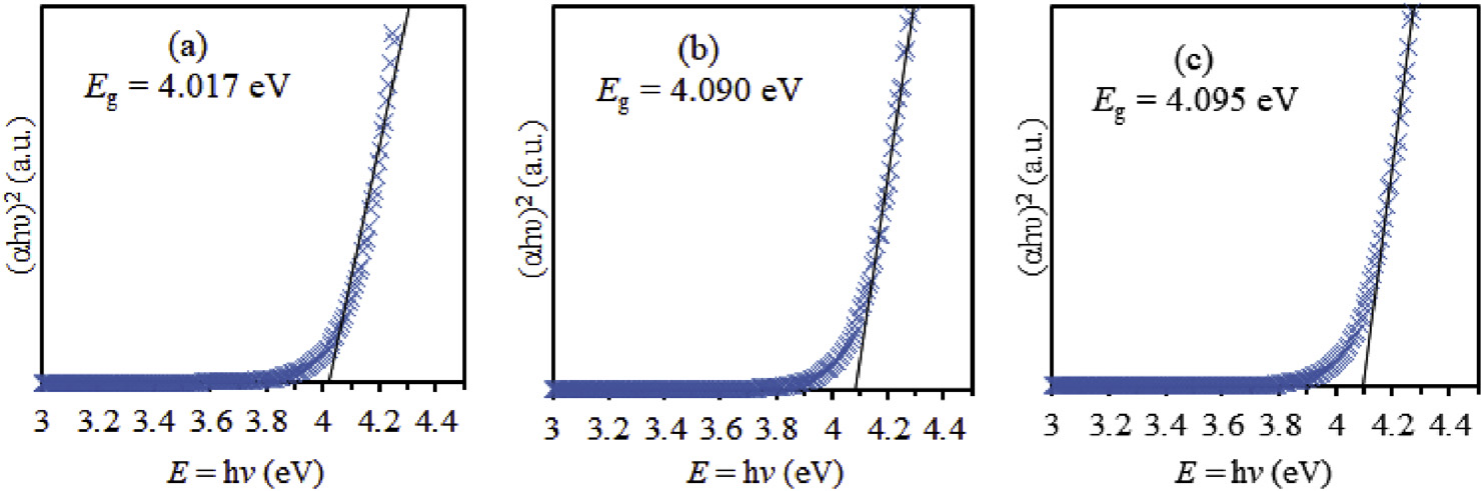
Tauc plots employed to estimate the optical band gap energy of (a): PVC-PS, (b): PVC-PS-5% silica, and (c): PVC-PS-10% silica thin films.

Tauc model gives the relationship of α and the incident photon energy (hv). In the high absorption region of semiconductor [39]:

(6) $${\left(\alpha hv\right)}^{1/m}=\beta \left(hv-E_{g}\right)$$

where β is a band tailing parameter, $E_{g}$ is the band gap energy, n is the power factor of the transition mode that depends on the nature of the material. The power number (n) exhibits 1/2 for allowed direct, 3/2 for forbidden direct, 2 for allowed indirect and ≥3 for forbidden indirect transitions. The Tauc formula can be rearranged as ln(αhv)=lnβ+m [40] to deduce the optical band transition mode of PVC-PS & PVC-PS-silica nanocomposite thin films. The power factor (m) that identifies the type of the optical transition mode can be calculated easily from the slope of ln(αhv) versus $\ln\left(hv-E_{g}\right)$ [40, 41]. The calculated value of *m* is found to be 1/2 indicating direct allowed transitions. Tauc plot is obtained by plotting the energy of the incident photon (hυ) in eV against $\;{\left(\alpha h\upsilon \right)}^{2}$*.* Figure 8 shows Tauc plot used to estimate the optical bandgap. The optical bandgap of PVC-PS is found to be 4.017 eV. As silica NPs are introduced in the nanocomposite thin films, optical band gap increases to 4.090 eV for PVC-PS-5% silica and to 4.095 eV for sample PVC-PS-10% silica. Optical band gap values are tabulated in Table 3.

#### 3.2.2 Band gap energy using the newly derived mathematical model

Accurate determination of film thickness and the optical bandgap from the transmittance and reflectance of the same samples under investigation is a target for several research groups. In this work, we propose a newly derived optical novel model using the measured transmission T(λ) to compute optical parameters such as, bandgap energy, film thickness, the difference in bonding–antibonding energy states, and the lifetime (τ) of the electrons of amorphous semiconductors and dielectric thin films. The probability of transition of an electron between two arbitrary states (|a and |b) per unit time ($W_{d\Omega }$) given that $E_{b}>E_{a}$ is calculated using the first-order time-dependent perturbation theory [42, 43],

(7) $$W_{d\Omega }=\frac{\pi e^{2}}{m_{e}}\frac{n_{\omega ,a}}{\omega }{\left|b|\vec{P}.{\vec{\varepsilon }}^{\left(a\right)}|a\right|}^{2}\rho_{\omega ,d\Omega }$$

where, $e,\;m_{e}$ and $\vec{P}$ are the charge, mass and momentum of the electron, respectively. The parameter $n_{\omega ,a}$ is the occupational number of photons and the polarization vector $\left({\vec{\varepsilon }}^{\left(\alpha \right)}\right),\;\;\text{where}\;\alpha =1,\;2$ describes the probable polarization phases of the photon. The factor $\rho_{\omega ,d\Omega }$ represents the number of allowed states in the frequency range ω and ω+dω,

(8) $$\rho_{\omega ,d\Omega }d\omega =\frac{\kappa^{2}d\kappa d\Omega }{{\left(2\pi \right)}^{3}}=\frac{\omega^{2}d\Omega }{{\left(2\pi c\right)}^{3}}d\omega$$

where, κ is the wave number. Eq. (8) is valid only if $\;E_{a}=\hbar \omega$. The momentum matrix in Eq. (7) is converted into the matrix of position using the momentum operator, $b\left|\vec{P}\right|a=im_{e}\omega b\left|\vec{x}\right|a$ [42]. The intensity $\left(I_{0}\left(\omega \right)\right)$ of the incident light can be written as,

(9) $$I_{0}\left(\omega \right)d\omega =n_{\omega ,a}c\hbar \omega \rho_{\omega ,d\Omega }d\omega =\frac{\hbar \omega^{3}n_{\omega ,a}d\Omega }{8\pi^{3}c^{2}}d\omega$$

Utilizing the sore mean of Eqs. (8) and (9), the transition probability rate $\left(W_{d\Omega }\right)$ in Eq. (7) becomes,

(10) $$W_{d\Omega }=\frac{8\pi^{2}}{3}\frac{e^{2}I_{0}}{\hbar^{2}c}{\left|b|\vec{x}|a\right|}^{2}$$

The energy absorbed per unit time (S(ω)) can be expressed as [43],

(11) $$S\left(\omega \right)=\frac{8\pi^{2}}{3\hbar c}e^{2}\omega I_{0}{\left|b|\vec{x}|a\right|}^{2}$$

The first-order time-dependent perturbation theory can then be employed to confirm the appearance of the damping factor as demonstrated by,

(12) $$\frac{\hbar^{2}\gamma }{2\pi }\frac{1}{{\left(E_{b}-E_{a}-\hbar \omega \right)}^{2}+\hbar^{2}\gamma^{2}/4}$$

and [43],

(13) $$\Phi \left(\omega \right)d\omega =S\frac{\hbar^{2}\gamma }{2\pi }\frac{1}{{\left(E_{b}-E_{a}-\hbar \omega \right)}^{2}+\hbar^{2}\gamma^{2}/4}d\omega$$

At infinite transition life time, γ vanishes, in the limit, γ→0, Eqs. (12) and (13) can be combined to yield,

(14) $$\frac{\hbar^{2}\gamma }{2\pi }\frac{1}{{\left(E_{b}-E_{a}-\hbar \omega \right)}^{2}+\hbar^{2}\gamma^{2}/4}\to \;\delta \left(E_{b}-E_{a}-\hbar \omega \right)$$

Therefore,

(15) $$\underset{\gamma \to 0}{\text{Lim}}\int \Phi \left(\omega \right)d\left(\hbar \omega \right)=S\left(\omega \right)$$

where, $\;\omega =\left(E_{b}-E_{a}\right)/\hbar$. The absorption coefficient α(ω) is defined by W. Heitler et al. as [43],

(16) $$\alpha \left(\omega \right)=\lim_{\Delta x\to 0}\left[-\frac{1}{I}\frac{\Delta I}{\Delta x}\right]=\frac{1}{I_{0}}\theta \Phi$$

where, Ө stands for the number of transitions in a very thin slab of thickness Δx.

The transmittance (T) and the coefficient od absorption α(ω) are explicitly related to each other [25],

(17) $$T=e^{-\alpha d}$$

where, *d* stands for the film's thickness. Using Eqs. (13), (16), and (17), the transmittance (T) can be written as,

(18) $$T=e^{-d*\theta \frac{4\pi \hbar e^{2}\omega }{3c}{\left|b|\vec{x}|a\right|}^{2}\frac{\gamma }{{\left(E_{b}-E_{a}-\hbar \omega \right)}^{2}+\hbar^{2}\gamma^{2}/4}}$$

**Figure 9:**
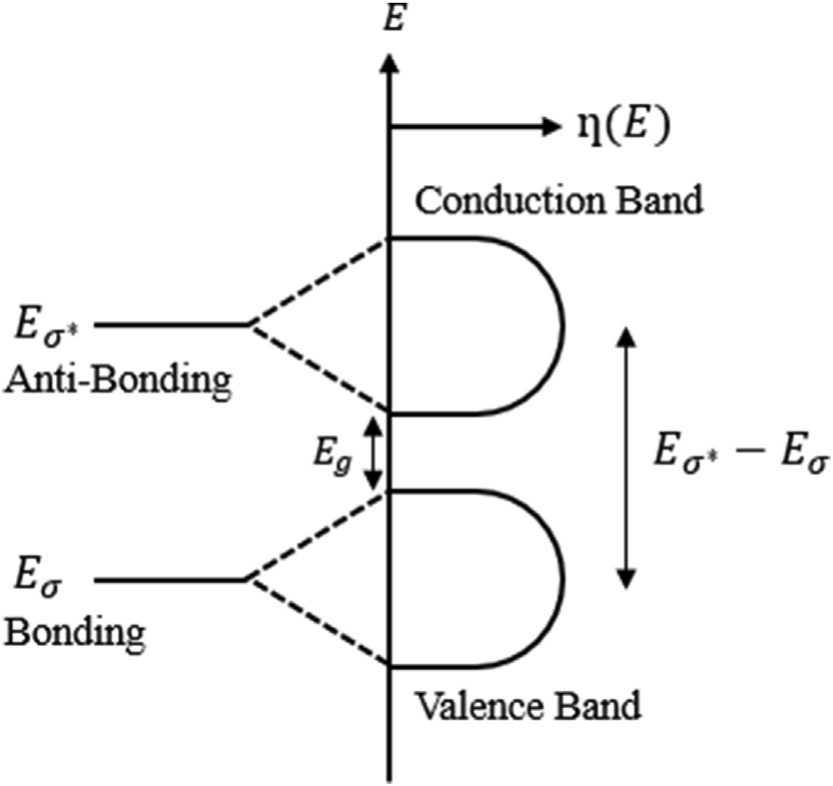
The molecular-orbital states (|σ and $\left|\sigma^{*}\right)$ are related to the energies $E_{\sigma }$ and $\;E_{\sigma^{*}}$ are broadening into the valence band and the conduction band when the solid is formed, where, η(E) represents the density of states while $E_{g}$ represents the band gap energy [44].

By using Eq. (18) and the absorption spectra of any amorphous semiconductor, we can determine the thickness of film and the optical constants simultaneously. Forouhi and Bloomer defined all parameters in Eq. (18) [44]. In tetrahedral coordinated covalent materials, linear combinations of atomic orbitals may lead to bonding (|σ) and antibonding ($|\sigma^{*}$) molecular states as shown in Figure 9.

The minimum value of the transmittance (T) occurs only when $\hbar \omega =E_{b}-E_{a}$ as demonstrated in Eq. (12). It is assumed that the maximum absorption occurs when $\;\hbar \omega =E_{\sigma^{'*}}-E_{\sigma^{'}}$, where $E_{\sigma^{'*}}$ and $E_{\sigma^{'}}$ are the energies in the conduction and valence bands such that $\;E_{\sigma^{'*}}=E_{\sigma^{*}}$, and $\;E_{\sigma^{'}}=E_{\sigma }$. This is attributed to the fact that, the densities of states (η(E)) are maxima throughout the conduction band and the valence band for $E_{\sigma^{'*}}$ and $E_{\sigma^{'}}$ as can be clearly seen in Figure 9. If $E_{b}=E_{\sigma^{'*}}$ and $E_{a}=E_{\sigma^{'}},$ then $\left|b=\right|\sigma^{'*}$ and $\left|a=\right|\sigma^{'}$. Hence, when $E_{\sigma^{*}}-E_{\sigma }=\hbar \omega ,$ the absorption is considered to be maximum [45].

The parameter θ is directly proportional to the number of filled states in the valence band ($\eta_{v}\left(E\right)f_{v}\left(E\right)dE)$ and the number of unoccupied states in the conduction band ($\eta_{c}\left(E'\right)\left[1-f\left(E^{'}\right)\right]dE')$ [27]. The functions, $\eta_{v}\left(E\right)$, $\eta_{c}\left(E'\right)$, and f(E) are the density of states in the valence, condition bands, and Fermi function, respectively,

(19) $$\theta \propto \int dE\int dE^{'}\eta_{v}\left(E\right)f\left(E\right)\eta_{c}\left(E^{'}\right)\times \left[1-f\left(E^{'}\right)\right]\delta \left(E^{'}-\left(E+\hbar \omega \right)\right)$$

Or

(20) $$\theta \propto \int \eta_{v}\left(E\right)f_{v}\left(E\right)\eta_{c}\left(E+\hbar \omega \right)\left[1-f_{c}\left(E+\hbar \omega \right)\right]dE$$

In the high-temperature limit, $f_{v}\left(E\right)$ ≈ 1 and $f_{c}\left(E+\hbar \omega \right)$ ≈ 0. Therefore,

(21) $$\theta \propto \int_{E_{bottom}-\hbar \omega }^{E_{top}}\eta_{v}\left(E\right)\eta_{c}\left(E+\hbar \omega \right)dE$$

where, $E_{top}$ stands for the valence band maximum and $E_{bottom}$ represents the conduction band minimum. The optical bandgap energy ($E_{g})$ is thus,

(22) $$E_{g}=E_{bottom}-E_{top}$$

As reported by [27],

(23) $$\eta_{v}\left(E\right)=const\times {\left(E_{top}-E\right)}^{p}$$

(24) $$\eta_{c}\left(E\right)=const\times {\left(E-E_{bottom}\right)}^{s}$$

Eq. (21) can be rearranged to become as:

(25) $$\theta =const\times {\left(\hbar \omega -E_{g}\right)}^{p+s+1}$$

If the valence band and the conduction band are parabolic so that p+s $=\frac{1}{2}$ , then,

(26) $$\theta =const\times {\left(\hbar \omega -E_{g}\right)}^{2}$$

In this is the case, the transmittance is then determined from Eqs. (13) and (26) to be:

(27) $$T=e^{-d\times const\;\frac{4\pi \hbar^{2}e^{2}\omega }{3c}{\left|\sigma^{'*}|\vec{x}|\sigma^{'}\right|}^{2}\;\frac{\gamma }{{\left(E_{\sigma^{'*}}-E_{\sigma^{'}}-\hbar \omega \right)}^{2}+\hbar^{2}\gamma^{2}/4}{\left(\hbar \omega -E_{g}\right)}^{2}}$$

Eq. (27) could be rewritten in the form of:

(28) $$T\left(E\right)=e^{-\;\frac{4\pi dE}{hc}\;\frac{A{\left(E-E_{g}\right)}^{2}}{E^{2}-BE+C}}$$

Eq. (28) can be rewritten to give T as a function of the wavelength (λ) as follows,

(29) $$T\left(\lambda \right)=e^{-\;\frac{4\pi d}{\lambda }\frac{A{\left(hc/\lambda -E_{g}\right)}^{2}}{{\left(hc/\lambda \right)}^{2}-B\left(hc/\lambda \right)+C}}$$

where, hc=1240eV, $A=const\frac{2\pi }{3}e^{2}\hbar^{2}{\left|\sigma^{'*}|\vec{x}|\sigma^{'}\right|}^{2}\gamma$, $B=2\left(E_{\sigma^{'*}}-E_{\sigma^{'}}\right)$, $C={\left(E_{\sigma^{'*}}-E_{\sigma^{'}}\right)}^{2}+\frac{\hbar^{2}\gamma^{2}}{4}={\left(E_{\sigma^{'*}}-E_{\sigma^{'}}\right)}^{2}+\frac{\hbar^{2}}{4\tau^{2}}$. Thus, T(λ) can be calculated in terms of five parameters [46]. Namely, the film thickness (*d)*, the optical band gap energy ($E_{g})$, the bonding-antibonding difference in energy states ($E_{\sigma^{'*}}-E_{\sigma^{'}}$), the lifetime (τ) of the electrons involved in the optical transitions and a quantity (*A*) which is related to the position matrix and τ. The parameters *A, B* and *C* fulfil the conditions, A>0, B>0, C>0, and $4C-B^{2}>0$. The previous conditions are necessary in order to determine n(E) from Kramers-Kronig relations. The advantage of this formulation lies in its ability to determine the thickness of film and the other four optical constants simultaneously.

**Figure 10:**
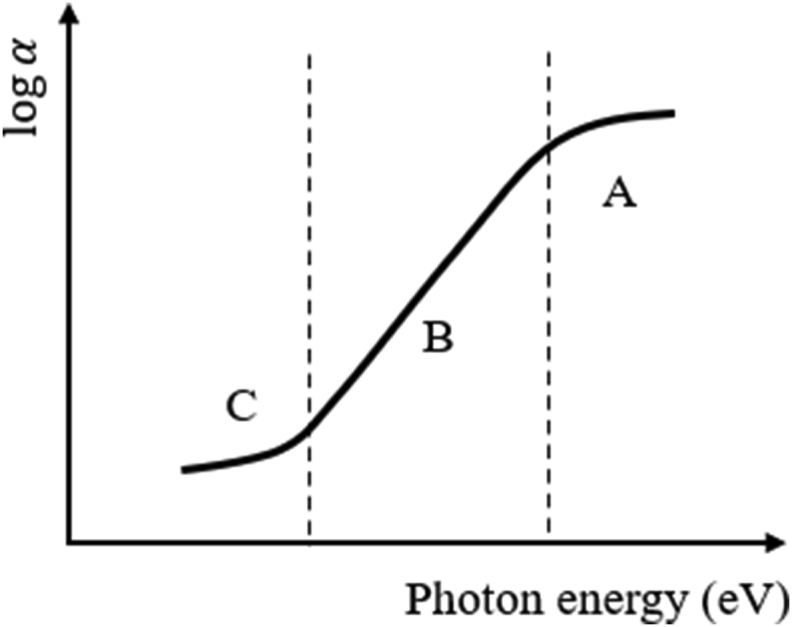
Three regions of the optical absorption coefficient of disordered materials.

**Figure 11:**
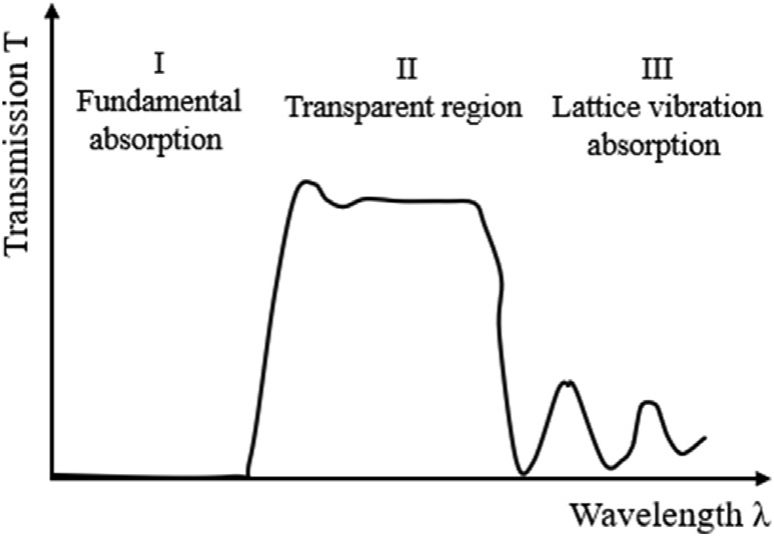
Schematic curve of transmission versus wavelength of an optical thin films [50].

Typically, the absorption edge of an amorphous semiconductor can be visualized in Figure 10. As shown in the figure, three distinct regions can be distinguished. Mainly, the high absorption region A ($\alpha \geq 10^{4}\;cm^{-1}$), the region of strong absorption, and the weak absorption tail C [47, 48, 49]. Figure 11 depicts schematically the typical curve of transmission versus wavelength of the optical thin films. Transparent region depends strongly on stoichiometry and purity of the material [50].

**Figure 12:**
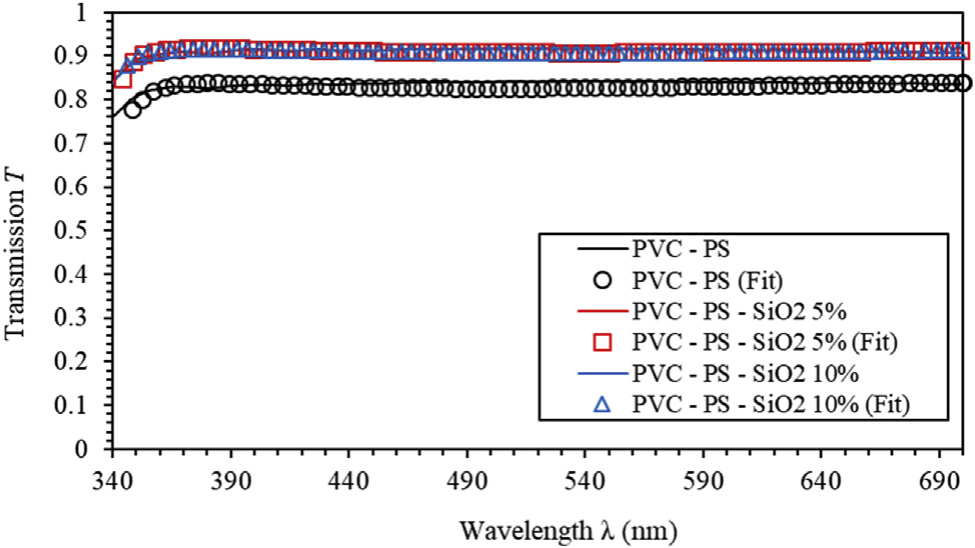
Transmission spectra of PVC-PS and PVC-PS-silica nanocomposite thin films with 5% and 10% concentrations of silica NPs. The experimental (solid lines) and model calculated (circle, square and triangle).

Figure 12 shows the transmission spectra of PVC-PS and PVC-PS-silica nanocomposite thin films with different concentrations of silica NPs. An excellent agreement can be readily seen between the experimental transmittance and the model fitted transmittance. Fitting parameters of the newly derived model applied to PVC-PS and PVC-PS-SiO_2_ NPs with different concentrations of SiO_2_ NPs thin films are given in Table 2. The band gap energy values PVC-PS and PVC-PS-SiO_2_ thin film were found to be 3.835 eV, 3.961 eV and 4.012 eV, respectively, with average thickness value of 300 nm. Incorporation of silica NPs into the polymer composites results in a noticeable increase in optical band gap as clearly shown in Table 2. Increasing SiO_2_ NPs doping level from 0% to 10% leads to a significant increase of *E*_*g*_ from 3.835 eV to 4.012 eV. The values of bandgap obtained from the newly derived model are in excellent agreement with those obtained from Tauc plot. The small deviation is attributed to the fact that the average thickness obtained from our model is slightly different from the real thickness obtained using UV-vis spectrophotometer.

**Table 2:** Fitting parameters of our newly derived model applied to PVC-PS and PVC-PS-SiO_2_ NPs with different concentrations of SiO_2_ NPs thin films.

Sample	$E_{g}$(eV)	d(nm)	A	B	C
PVC-PS	3.835 ± (0.01687)	292.597 ± (5.1412)	0.0319 ± (5.14E-6)	8.057 ± (0.0010)	16.137 ± (0.0208)
PVC-PS- 5% SiO_2_	3.961 ± (0.01118)	306.035 ± (4.1524)	0.0351 ± (4.79E-6)	11.451 ± (0.0012)	28.633 ± (0.0424)
PVC-PS- 10% SiO_2_	4.133 ± (0.01547)	302.351 ± (7.1551)	0.0372 ± (2.48E-6)	13.757 ± (0.0016)	29.351 ± (0.0357)

#### 3.2.3 Urbach energy

A deeper insight into understanding the amorphous and low crystalline thin films might be accomplished by computing the Urbach energy, $E_{U}$ [37, 52]. It is well known that $E_{U}$ provides details of the transitions between valence band and localized states of the conduction band [47, 48, 49]. Near the optical band edge, the relationship between (α) and (hv) can be expressed empirically as,

(30) $$\alpha =\alpha_{0}\;\exp\left(hv/E_{U}\right)$$

where $\alpha_{0}$ is a constant, hv is the incident photon energy and $E_{U}$ is the Urbach energy. By plotting ln(α) versus the photon energy, it is possible to determine $\;E_{U}$. Urbach energy values of the investigated samples are tabulated in Table 3. Urbach energy of PVC-PS thin film is found to be 356meV. Our results indicate that the values of Urbach energy of thin films increase as the concentration of silica into PVC-PS thin films increases. The increase in the Urbach energy values upon introducing silica NPs into PVC-PS thin films indicate higher disorder in the films that could be attributed to the highest surface interaction induced [4]. Furthermore, Urbach suggested another formula correlate the absorption coefficient (α) with the optical energy gap, $\alpha =\beta \;\exp\left[\sigma \left(hv-E_{g}\right)/k_{B}T\right]$ [20], where β is a pre-experimental constant, $k_{B}T$ is the product of the Boltzmann constant and the temperature and given by (0.0259eV) at T=300K and σ is the steepness parameter. This parameter was initially suggested by Skettrup [53] is given by $\sigma =k_{B}T/E_{U}$. Parameter σ is also related to the strength of the electron-phonon interaction ($E_{e-p})$ by $E_{e-p}=2/3\sigma$ [37, 54]. The obtained values of σ and $E_{e-p}$ are given in Table 3. Our results indicate that σ and $E_{e-p}$ of PVC-PS-SiO_2_ NPs thin films are inversely proportional. This relationship can be explained in terms of the alteration of ionicity and the anion valence as silica nanoparticles are dispersed into polymeric matrix [55, 56]. The increase in $E_{e-p}$ with silica concentration contributes significantly to the augmentation of the filled bands [57].

**Table 3:** Band gap energy, Urbach energy and electron-phonon interaction of PVC-PS, PVC-PS-SiO_2_ 5% and PVC-PS-SiO_2_ 10% thin films.

Parameter	Symbol	PVC-PS	PVC-PS-5%silica	PVC-PS-10%silica
Average Thickness (nm)	d	300	300	300
Thickness determined using our model (nm)	*d*	292.597	306.035	302.351
Band gap energy from Tauc plot (eV)	$E_{g,Tauc}$	3.993	4.002	4.003
Band gap energy from our model (eV)	$E_{g}$	3.835	3.961	4.133
Urbach energy (meV)	$E_{U}$	356	1067	1066
Steepness parameter (eV^−1^)	σ	0.0726	0.0242	0.0243
Electron-phonon interaction (eV)	$E_{e-p}$	9.183	27.548	27.435

### 3.3 X-ray diffraction analysis

**Figure 13:**
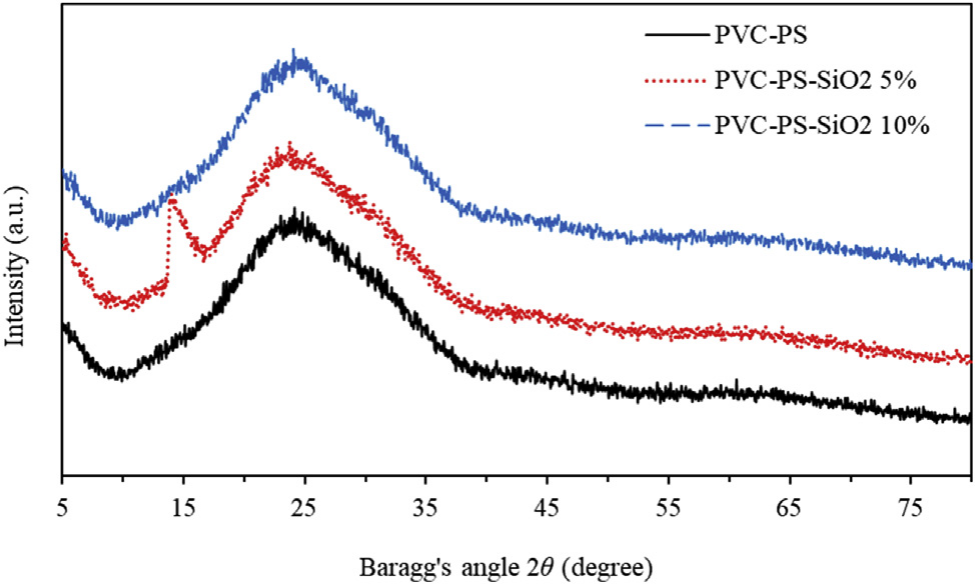
The XRD patterns of PVC-PS and PVC-PS-silica nanocomposite thin films with 5% and 10% concentrations of silica NPs.

The crystalline nature of PVC-PS and PVC-PS-silica nanocomposite thin films with 5% and 10% of silica NPs is examined by means of Powder X-Ray Data Analysis System using Cu*Kα* ray with a wavelength of 0.1540598 nm as illustrated in Figure 13. As can be seen, no discrete or sharp diffraction peaks are observed. The amorphous nature of PVC-PS nanocomposite thin film is confirmed by the broad peak in the region of 15°–35° [58]. The silica has an amorphous nature [59, 60], therefore, addition of 5% and 10% of silica NPs does not fundamentally alter the degree of crystallinity of the doped polymerized thin films. The XRD spectra of the three investigated samples are identical. This is consistent with the findings of A. Abdelghany *et. al* and T. Abdel-Baset *et. al* studies [10, 61].

### 3.4 Surface analysis studies

#### 3.4.1 Scanning Electron Microscopy measurements

**Figure 14:**
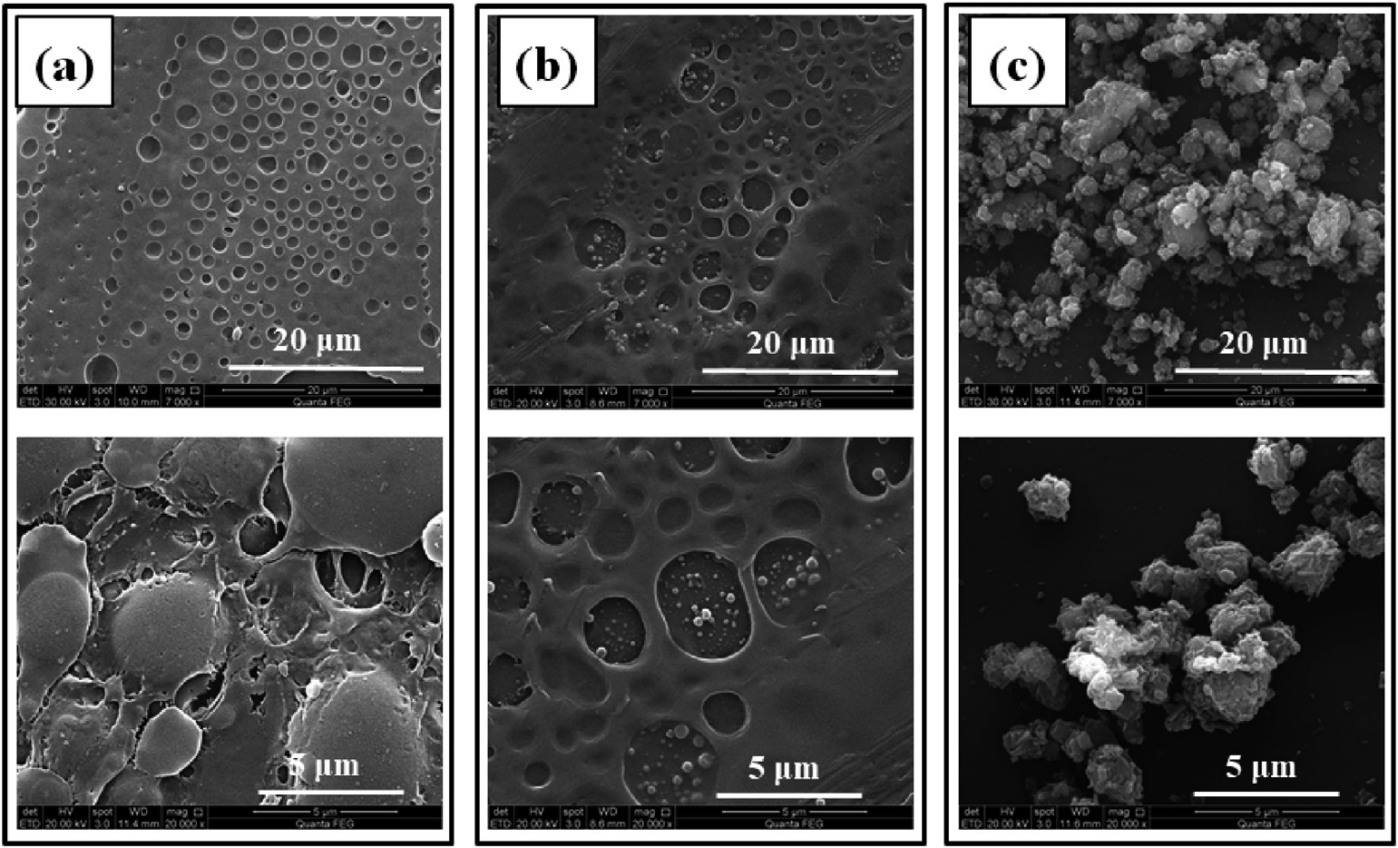
The SEM micrographs of (a) PVC-PS, (b) PVC-PS- 5% silica and (c) PVC-PS- 10% silica nanocomposite thin films.

The surface morphology of thin films is inspected using Scanning Electron Microscopy (SEM). Surface morphology of PVC-PS, PVC-PS-SiO_2_ 5% and PVC-PS-SiO_2_ 10% thin films at 20 μm and 5 μm magnifications are presented in Figure 14. Figure 14-(a) shows that the nanocomposite thin films of PVC-PS exhibit an amorphous nature consistent with XRD patterns. Figure 14-(b) and 14-(c) show the silica NPs on the surface immersed into the thin film matrix. The observed silica NPs dimension is within the average particle size between (100–400) nm in diameter. Furthermore, SEM was used to examine the morphology and dispersion of silica NPs on the surface of PVC films. The SEM images show a good dispersion of silica NPs on the surface of the PVC-PS films. This provides substantial evidence of the validity of our synthesis process of obtaining nano-silica.

#### 3.4.2 Contact angle measurements

**Figure 15:**
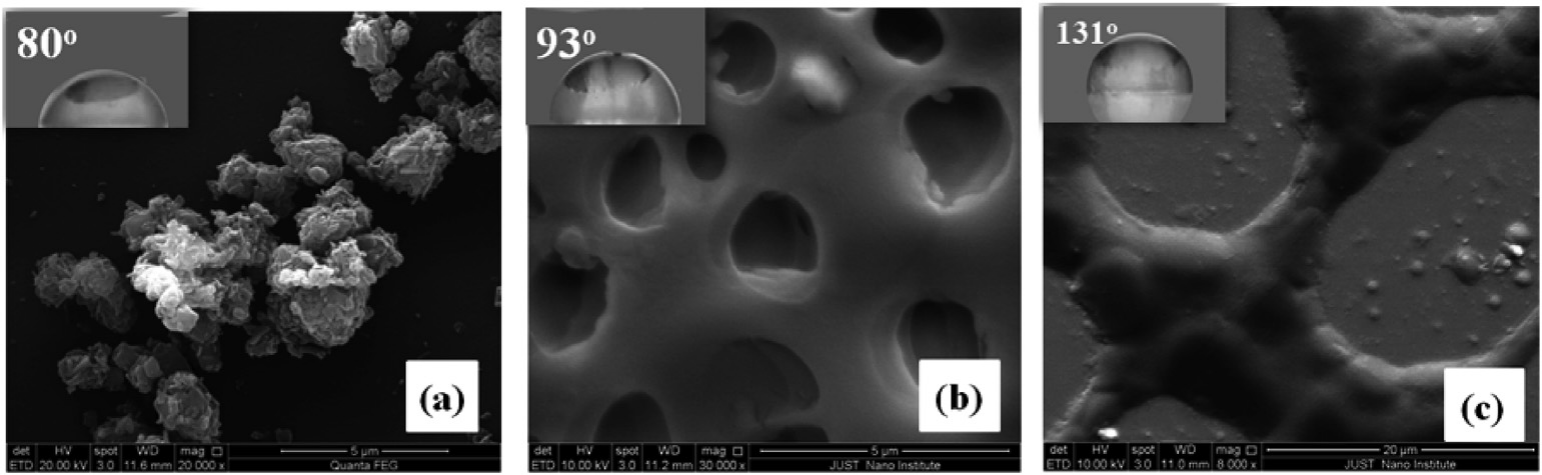
SEM images and the WCAs (inset) of PVC-PS-SiO_2_ nanocomposite films on a glass substrate; (a) as-prepared, and annealed at (b) 100 °C and (c) 200 °C.

In order to perform accurate water contact angle (WCA) measurements, the PVC-PS-10% silica nanocomposite thin films were chosen and annealed in air at various temperatures (100, 200, 300, 400 and 500) ^o^C for 1 h. By doing so, we can ensure that the PVC-PS-10% silica nanocomposite thin film has relatively high concentration of silica nanoparticles. The observed surface transformation to hydrophobic is attributed to the presence of high concentrations of silica nanoparticles inside PVC-PS polymeric matrix. Furthermore, PVC-PS-10% silica nanocomposite thin films were found to have the highest transmittance indicating their hydrophobicity and anti-reflectance dominant features. Water contact angles (WCA) were measured at room temperature. The corresponding surface wettability of the as-synthesized samples was studied by measuring the WCA using a water droplet (pH = 7) of 10*μ*L. The WCA values of the glass treated as-synthesized samples with annealed at (100, 200, 300, 400 and 500 °C) for 1 h were compared with the as-prepared sample. It is customary that the solid surfaces with contact angles over 120^o^ are super-hydrophobic. Figure 15 shows the SEM images of dip-coated PVC-PS-10% silica nanocomposite films. The water droplets on the surfaces are shown in the inset as a guide for eyes. Figure 15-a, b display the SEM images of films as prepared and that of films annealed at 100 °C. Obviously, both exhibit a good dispersion of silica NPs on the surface of the PVC-PS films with island structure. The observed WCAs were 80^o^ and 93^o^ for as-prepared thin films and films annealed at 100 °C, respectively. Figure 15-c shows the SEM image of the films post-annealed at 200 °C. Post-annealed film surfaces exhibit an island expansion indicate strong hydrophobicity nature with WCA of 131^o^. The post-annealed thin films were found to have suitable wax-like nanostructure surfaces that make them potential candidates for hydrophobicity applications. The melting points of PVC and PS are 260 °C and 240 °C, respectively. Thus, the films were annealed at a temperature not to exceed 200 °C. This observation is in excellent agreement with the finding of A. Sriboonruang *et. al* study [11].

## 4 Conclusions

In summary, PVC-PS-silica nanocomposites in the form of thin films with silica nanoparticles contents ranging from 0 to 10 wt. % were synthesized. Smooth films were obtained by allowing the solvent to evaporate overnight at 25 °C. Films microstructure were investigated using XRD. All samples were found to exhibit an amorphous structure. SEM micrographs indicate that the morphology of the surfaces of as prepared thin films depends on the level of concentration of silica nanoparticles with an average particle size (100–400) nm. In addition, SEM was used to examine the morphology and dispersion of silica nanoparticles on the surface of PVC-PS thin films indicating a good dispersion of silica on the surface of the PVC-Ps films. This proves the validity of our synthesis process for obtaining nano-silica. Additionally, we found that the hydrophobic nature of the thin films might be controlled by adjusting the post-annealing temperature. Our results indicate that thin film surface transforms to hydrophobic when the film was annealed at 200 °C with water contact angle of 131^o^.

The main theme of this work is the introduction of a new model utilizing the experimental transmittance data as a function of photon wavelength (T (λ)) to characterize and interpret all of the optical properties of the as prepared thin films. The optical properties of thin films were characterized by measuring their transmittance and reflectance. The index of refraction, extinction coefficient and optical band gap have been calculated accordingly. The transmittance of PVC-PS thin film was found to be on average of around (>80%) in the visible region. The transmittance was found to increase by introducing SiO_2_ nanoparticles into PVC-PS polymeric thin films. The relevant optical properties were significantly affected accordingly. Index of refraction of the PVC-PS thin film was found in the range between 1.8 and 2.2 and found to decrease as the SiO_2_ nanoparticles concentration was introduced. Optical band gap energy was calculated by two methods, namely, Tauc plot and the newly derived mathematical model. The values of optical band gap of PVC-PS-SiO_2_ nanocomposite thin films with different concentration of SiO_2_ NPs were calculated using the three models were found to be in good agreement. The slight differences between the values of optical band gap obtained from the two models could be attributed to the slight difference in the values of the average thickness obtained using the three different models. Using the newly derived model, optical band gap of PVC-PS thin film was found to be 3.835 eV. Our results indicate a slight increase in the value of the optical band gap upon introducing SiO_2_ NPs in the polymer composite thin films. The superiority of the newly derived model lies in its ability to determine the optical bandgap of low crystalline composites by accurate fitting of their own transmittances. On the contrary, other existing optical models demand the usage of transmission data of other amorphous composites to estimate the optical bandgap and film thickness of thin films under investigation. Furthermore, experimental determination of optical bandgap by exploiting the polarized light in spectroscopic elliposmetry data analysis requires strict accurate determination of the film thickness at hand which is not the case when employing the current derived model. Secondly, in order to overcome the correlation between different optical constants of the composites, an individual measurement of each optical constant has to be conducted separately. Interestingly, the new derived model guarantees the simultaneous accurate measurement of film thickness and optical band gap. Furthermore, implementing the newly derived mathematical model enables the determination of other optical constants such as bonding–antibonding difference in energy states, the lifetime of the electrons involved in the optical transitions and a factor that depends on the position matrix and electrons lifetime can be calculated directly by our five parameters dependent model. The predictions of the newly proposed model have been found to agree fairly well with findings of other well-known methods such as Tauc plot methods.

## Declarations

### Author contribution statement

Qais M. Al-Bataineh, A. M. Alsaad: Conceived and designed the experiments; Performed the experiments; Analyzed and interpreted the data; Contributed reagents, materials, analysis tools or data; Wrote the paper.

A. A. Ahmad, Ahmad Telfah: Conceived and designed the experiments; Performed the experiments; Analyzed and interpreted the data.

### Funding statement

This work was supported by 10.13039/501100004035Jordan University of Science and Technology (282/2019).

### Competing interest statement

The authors declare no conflict of interest.

### Additional information

No additional information is available for this paper.
